# Impacts of recent climate change on crop yield can depend on local conditions in climatically diverse regions of Norway

**DOI:** 10.1038/s41598-023-30813-7

**Published:** 2023-03-03

**Authors:** Shirin Mohammadi, Knut Rydgren, Vegar Bakkestuen, Mark A. K. Gillespie

**Affiliations:** 1 Department of Environmental Sciences, Western Norway University of Applied Sciences, 6856 Sogndal, Norway; 2 Department of Plant Science, Norwegian University of Life Sciences, 1433 Ås, Norway; 3 Norwegian Institute for Nature Research, Sognsveien 68, NO-0855 Oslo, Norway

**Keywords:** Ecology, Agroecology, Climate-change ecology

## Abstract

Globally, climate change greatly impacts the production of major crops, and there have been many attempts to model future yields under warming scenarios in recent years. However, projections of future yields may not be generalisable to all crop growing regions, particularly those with diverse topography and bioclimates. In this study, we demonstrate this by evaluating the links between changes in temperature and precipitation and changes in wheat, barley, and potato yields at the county-level during 1980–2019 in Norway, a Nordic country with a range of climates across a relatively small spatial scale. The results show that the impacts of climate variables on yield vary widely by county, and that for some crops, the strength and direction of the link depends on underlying local bioclimate. In addition, our analysis demonstrates the need for some counties to focus on weather changes during specific crucial months corresponding with certain crop growth stages. Furthermore, due to the local climatic conditions and varying projected climate changes, different production opportunities are likely to occur in each county.

## Introduction

Food security and eliminating hunger are fundamental to fulfilling the global sustainable development goals^1^, but sensitivity to climate change has made food production challenging^2^. Extreme weather events such as heatwaves, droughts, and heavy and prolonged precipitation have increased in recent decades^3^, with significant negative impacts on agricultural production^4,5^. According to assessments by the Food and Agriculture Organization of United Nations (FAO), climate-related challenges are one of the leading factors of food insecurity^6^, causing yield reduction for major crops globally during the last 20 years^7–9^. This situation will become more critical as the current warming trend predicts average global temperature increases of 1.5–4.8 °C by 2100^9^.

During the last 20 years, the fluctuation of crop production has contributed to a strong research focus on evaluating the impact of climate change on crops at national and regional levels. These studies show that impacts vary by local climatic conditions, crop, soil types, geography, management system, and technological application^10,11^. In regions at low latitudes, temperatures may exceed the optimum threshold for crop productivity with only a slight increase of local temperature (1–2 °C), subsequently decreasing yield due to heat and drought stresses^8,12,13^. Conversely, at mid to high latitudes, local mean temperature rises of 1–3 °C may improve production by providing optimum temperatures for a longer growing season^14,15^. Therefore, mid to high latitude regions may be well placed to adapt agricultural production to climate change, with moderate temperature increases potentially providing opportunities to increase crop production^13^. However, such generalisations are unlikely to apply to all countries, especially those with large spatial variation in climate^16–18^, and more studies are required to make projections for a wider range of geographical areas.

In particular, global and regional climate impact projections of future yields need to be complemented with finer-scale local assessments, particularly in countries with a diverse topography and bioclimate, such as Norway. The mountainous topography and large latitudinal range of this country provide several types of climate over small spatial scales, with strong regional oceanity and temperature gradients^19,20^. This diversity influences land use, as 49% of agricultural area is concentrated in south-eastern counties with more continental and warmer climates^21^, and other arable patches are distributed throughout the oceanic west, and mountainous central regions. While annual mean temperature in Norway is projected to increase by 1.6–4.5 °C and precipitation by 3–14% (concentration scenarios RCP2.6 and RCP8.5) by 2100, sub-national expectations vary widely^22^. For example, northern areas of the country are expected to experience the largest increases in annual mean temperature, while western areas are likely to experience the smallest temperature change but greatest increase in annual precipitation^22^. With many crops at their northern limit for cultivation in Norway, these changes may increase the growing season length by up to 2 months in some regions^22^, increase the availability of suitable land and enhance the potential for improving yield by exploiting future climatic changes^23^. However, assessments in neighbouring countries, such as Denmark, Sweden and Finland, suggest that the response of annual crops to climate change in Nordic countries may be difficult to project: increasing temperature may improve opportunities or reduce productivity by exceeding optimum thresholds^16,18,24,25^. Similarly, increased precipitation may reduce the need for irrigation, but excessive precipitation may also negatively impact productivity^14,25^, particularly if it occurs as infrequent and concentrated heavy downpours. It is therefore important to complete an assessment for Norway, particularly given the wide range of climatic conditions found throughout the country.

In this study, we analyse publicly available crop yield and weather datasets to statistically evaluate the effect of changes in temperature and precipitation on wheat, barley, and potato yield during 1980–2019. We first aimed to understand how yield of the three crops may have been affected by climate changes in the main crop growing regions of South Norway during the study period (crops are not grown in large quantities in northern Norway), by assessing whether growing season temperature and precipitation could provide adequate predictors of yield changes. This is a common empirical approach in assessing past climate impacts on yield^12,26^. In line with findings from other Nordic countries^18,28^, we generally expected yield increases to be positively related to increases in temperature. However, to take account of the range of climates in Norway, we incorporate bioclimatic gradients in our statistical modelling and assess the degree to which trends can be generalised across the country. We hypothesised that temperature and precipitation would have differential effects on yield among the main crop growing counties, with positive effects of temperature in the cold-limited mid-Norway counties, and negative effects of precipitation in the humid western counties. Furthermore, in some recent studies, it was shown that pooling or averaging climate variables over the entire growing season could mask statistical modelling effects on finer timescales and that using monthly climate variables could provide useful alternative predictors of crop yield^26,29^. Our second aim was therefore to explore whether this is the case for Norwegian counties by separately assessing county yield relations with monthly weather variables, to identify the factors that best explain yield changes. We anticipate that these findings will assist farmers, agribusiness, and policymakers develop adaptation measures and better mitigate the effects of future climate changes.

## Method

### Data collection

Yield data were used for all the counties of South Norway (Supplementary Table S1) for barley (*Hordeum vulgare* L.), wheat (*Triticum aestivum* L.) and potato (*Solanum tuberosum* L.) from Statistics Norway^21^. We used annual production and harvested area of each crop during 1980–2019 to calculate crop yield (kg/decare (1 decare = 0.1 hectare)) by dividing production by area in each county. Some counties did not grow all three crops in some years (Supplementary Table S2), and only counties with data for 25 years or more were selected for data analysis.

We used daily temperature and precipitation data for the crop growing season, which we define as the months of May, June, July, and August. This tends to be the period when growing conditions are most suitable for crops in Norway, although this can vary across counties and years^30^. Note also that the length of the required growing season for each crop is different, and various crops may use the entire length of this period (perennial crops) or part of it (annual crops). Diagrams indicating the growth phases of each crop during the growing season can be found in the supplementary material (Fig. S1). Weather data were collated from the Norwegian Meteorological Institute^31^ using meteorological stations with a record of at least 35 years (Supplementary Table S3). Additional criteria for station selection were proximity to the agricultural area and elevation below 200 m a.s.l. (assumed to be the maximum elevation for the target crops). To ensure good temporal and spatial coverage of data, each county was represented by data from 2 to 5 stations. Where available, we used both minimum and maximum temperature from each station, and then calculated average daily temperature as the mean of these two. We summarised weather data for the growing season by (1) averaging daily temperature and precipitation values across the stations for each county, and then (2) averaging the county-level daily temperature values (to derive annual minimum, maximum and average temperatures), and summing the precipitation data. For our initial analysis (see “Data analysis” section) we used only average temperature (as minimum and maximum temperature are highly correlated) and summed precipitation as explanatory variables. For our exploratory analysis (see “Data analysis” section), we were able to use all four of the growing season variables, as well as their monthly counterparts. For these, we repeated step (2) above, but across each month of the growing season.

### Bioclimatic gradients

We used two gradient variables to characterize the bioclimate of each county in Norway. These variables were derived from a gridded climate dataset at a resolution of 1 km pixel size for all mainland Norway^32^. The dataset comprises fifty-four climatic, topographical, hydrological and geological variables collated from a range of sources and represented in a GIS stack. A full list of variables of all types are detailed in Bakkestuen et al.^20^. The GIS stack was exported to a matrix, normalized and standardized, and then subjected to a PCA (Principal Component Analysis) ordination revealing four environmental gradients^20^. In this study, we use the first two of these PCA gradients: (1) regional variation from coast to inland and from oceanic/humid to continental areas, with high scores corresponding to regions further from the coast/ocean and cooler winter temperatures, and (2) regional variation from north to south and from high to low altitudes, with high scores corresponding to areas with low elevation, less rugged terrain and warmer summer temperatures. The two variables not chosen for this study related to solar radiation and fine-scale terrain relief; these were omitted to minimize the complexity of models and they were considered less important to the research questions. The two chosen PCA axes corresponded to the two bioclimatic gradients often used in expert classifications of Norway into biogeographical regions: vegetation “sections” (from highly oceanic to slightly continental) and vegetation “zones” (from nemoral to alpine zones; Ref.^19^). We hereafter refer to these PCA axes as “inland” and “altitude” gradients, respectively. To calculate a county-level value for each of the two bioclimate gradients, we calculated mean values for each arable land parcel, and then calculated overall mean values for each county. We used the CORINE 2018 Land Cover map data to identify arable land areas^33^, and zonal statistics were calculated in QGIS (version 3.18.1—Zurich^34^).

### Data analysis

The R programming environment (version 4.2.2; https://cran.r-project.org/) was used for all analyses^35^. Firstly, to assess trends in weather variables and crop yields over the 40 year period, we performed linear regression with *year* as a continuous explanatory variable. We then performed two types of modelling: (1) linear mixed effects modelling (LMM) to assess the overall combined impact on yield of growing season temperature and precipitation trends and bioclimatic gradients, and (2) County-level LASSO (Least absolute shrinkage and selection operator) linear regression models to explore the impacts of monthly and growing season weather trends.

For both of these models types, we initially detrended all time series data. As crop yield changes over time may result from a combination of factors, it is common to detrend yield data to remove effects of technological advances^36^. Furthermore, when regression predictor terms are also time series, more reliable models are achieved by detrending both response and predictor variables^37^. For our yield, temperature and precipitation data, we explored detrending by (a) first difference, (b) linear trend removal (using residuals of linear regression of the variable against *year*), (c) non-linear trend removal (as (b) but using cublic spline additive models) and (d) including *year* as a covariate in models with non-detrended variables. In all cases, trends were found to be linear, stationary (KPSS test, p > 0.05) and with no unit root (PP-test, p < 0.05), suggesting that method b) linear trend removal, was appropriate. We further visually checked autocorrelation function plots, which confirmed that this method adequately removed the temporal autocorrelation for all variables.

The linear mixed-effects models (one for each crop) were built with the interactions between temperature and the two bioclimatic gradient variables (from the PCA analysis described above), and between precipitation and the two bioclimatic gradient variables:

$$\Delta y= \Delta T+ \Delta P+I+A+ \Delta TI+\Delta TA+ \Delta PI+ \Delta PA$$

where Δ*y* is the detrended yield (kg/decare), ΔT is detrended average temperature (°C), ΔP is detrended summed precipitation (mm), and where *I* (*Inland*) and *A* (*Altitude*) are the bioclimatic gradients. The slope coefficients from these models can be difficult to comprehend given that both yield and weather variables are expressed as deviations from annual trends. However, as discussed by Lobell et al.^36^, they can be assumed to indicate that positive (or negative) coefficients represent a positive (or negative) association with crop yield. In addition, to account for any residual county level differences in weather effects, the models were fitted with random slopes for temperature and precipitation for each county. Random intercepts were unnecessary because all intercepts were reduced to zero due to the detrending process. Models were fitted using the *glmmTMB* package^38^. We checked that the models did not suffer from collinearity by inspecting variance inflation factors using the *performance* package^39^. The validity of all models was checked graphically for the assumptions of residual normality and homoskedasticity using the *DhARMA* package^40^. We further checked the performance of these models by performing leave-one-out cross validation, where a single year is removed from the dataset, the model is re-run on the remaining data and predictions are made for the removed year. This was repeated for every year of the dataset and predictions were compared to observations using the root square mean error (RMSE).

Our second modelling approach was conducted for two reasons. First, the performance of the mixed effects models were generally low (see “Results”), which we contribute to the possibility that they failed to account for county-specific weather effects on finer temporal scales. Second, the novel mixed effects models did provide some novel insights into weather-yield relations across varying bioclimatic gradients, and we wanted to assess these findings on finer scales. We therefore constructed LASSO models using the *glmnet* package^41^. This modelling procedure provides a method to explore the importance of a large number of collinear explanatory variables, avoiding issues of overfitting and multicollinearity. As our monthly and seasonal minimum, maximum and average temperatures are strongly correlated, this procedure provides an opportunity to identify the monthly or seasonal predictors that are likely to be most important to yield changes at the county level. The method estimates a regularisaton parameter, λ^42^, to control the shrinkage of less important predictors to zero, and this is optimised via cross-validation^43^. Importantly for this study, the LASSO model is typically designed for prediction rather than inference, and we only use the procedure here as an exploratory tool, indicating levels of uncertainty in coefficient estimates using bootstrapped confidence intervals^44^. The LASSO models were fitted to the detrended response variables (yield of wheat, barley and potato) for each county separately. We used the detrended predictors: minimum, maximum and average temperature for each growing season month (May to August), and for the entire growing season for comparability to the mixed effects models, and summed precipitation for each growing season month and the entire season. All variables were standardised to make coefficients comparable. We used the leave-one-out approach in initial cross-validation to obtain optimal λ values^45^. Values of λ that minimized the Mean Square Error were taken as optimal, and we subsequently assessed residuals using the *plotres* function of the *plotmo* package^46^. Finally, coefficients were bootstrapped with 999 replicates using the *bootLasso* function of the *HDCI* package^47^. All models (Linear mixed models and LASSO models) were repeated with the data for 2018 removed. This year was abnormally warm and dry, and was a clear outlier in many of the residual plots. It was therefore removed from all models for consistency and to explore how one extreme year impacted weather-yield relations. Coefficients and 95% Confidence Intervals (CIs) for both models with and without 2018 are presented.

## Results

### Yield and climate trends

**Figure 1:**
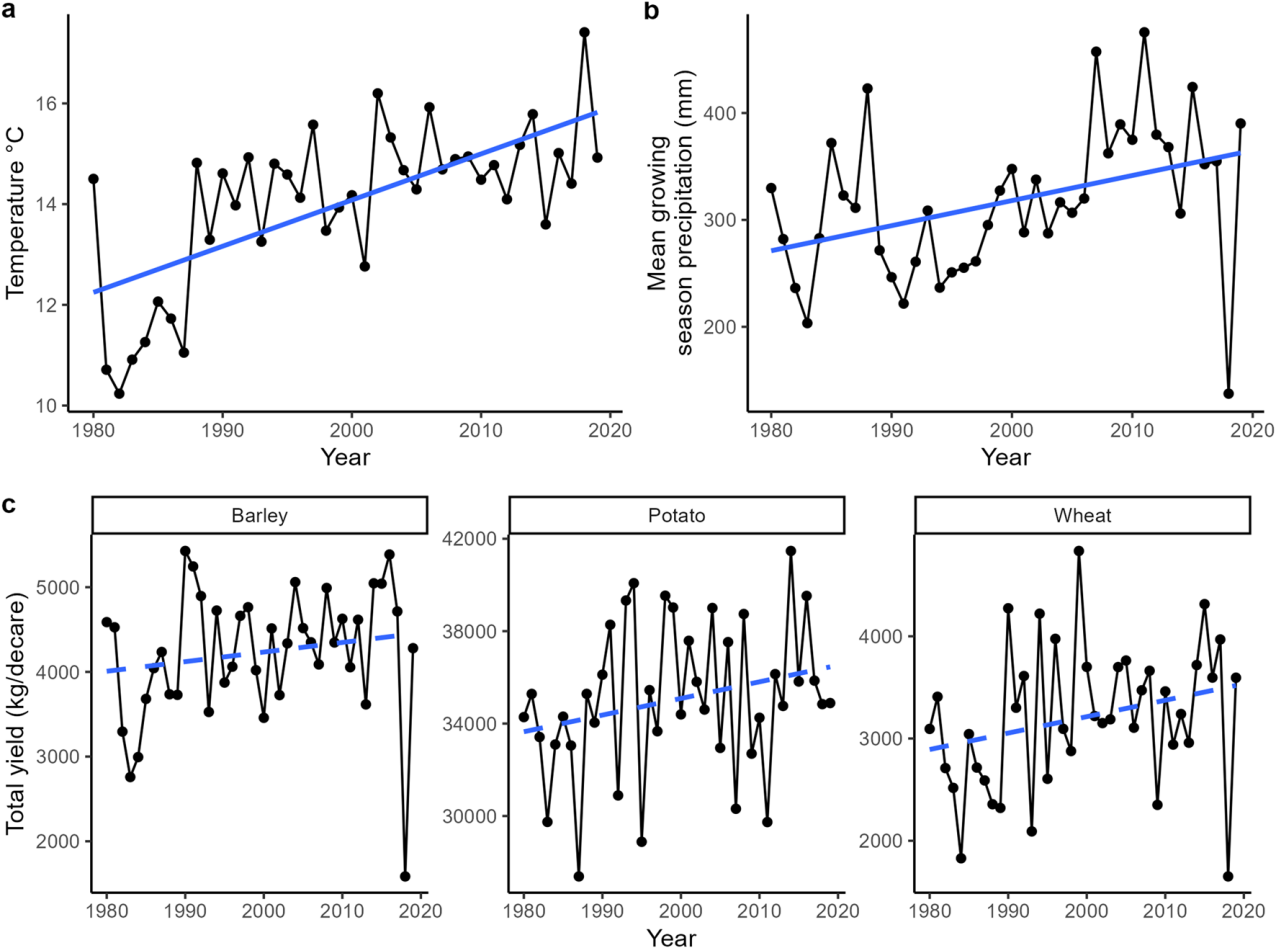
Country-level time series of (**a**) mean growing season temperature, (**b**) mean growing season precipitation and (**c**) total yields of the three study crops. Solid blue lines are significant (p < 0.05) linear regression lines. Dotted lines are non-significant linear trend lines.

Over the 40 year study period, agricultural areas in South Norway have experienced a steady increase in mean growing season temperatures (slope = 0.09 °C ± 0.02 SE, p < 0.001; Fig. 1a) and total rainfall (slope = 2.35 mm ± 0.89 SE, p = 0.012; Fig. 1b). The yields of the three main crops showed an increasing tendency, although none of the trends were significant at the country level (Fig. 1c). 2018 stands out as a particularly dry and warm year, with average temperatures of 17.4 °C and precipitation at 137.5 mm, and relatively low levels of wheat and barley yields.

At the county level, yields of the three crops showed a wide range of trends (data not shown). As with the country-level trends there was a high degree of inter-annual variation, although the wheat trends appear relatively consistent across the eight counties included in the analysis. By contrast, barley and potato yields exhibit widely differing trends among the counties, including strong increases and decreases over the study period.

### Bioclimate gradient scores

**Figure 2:**
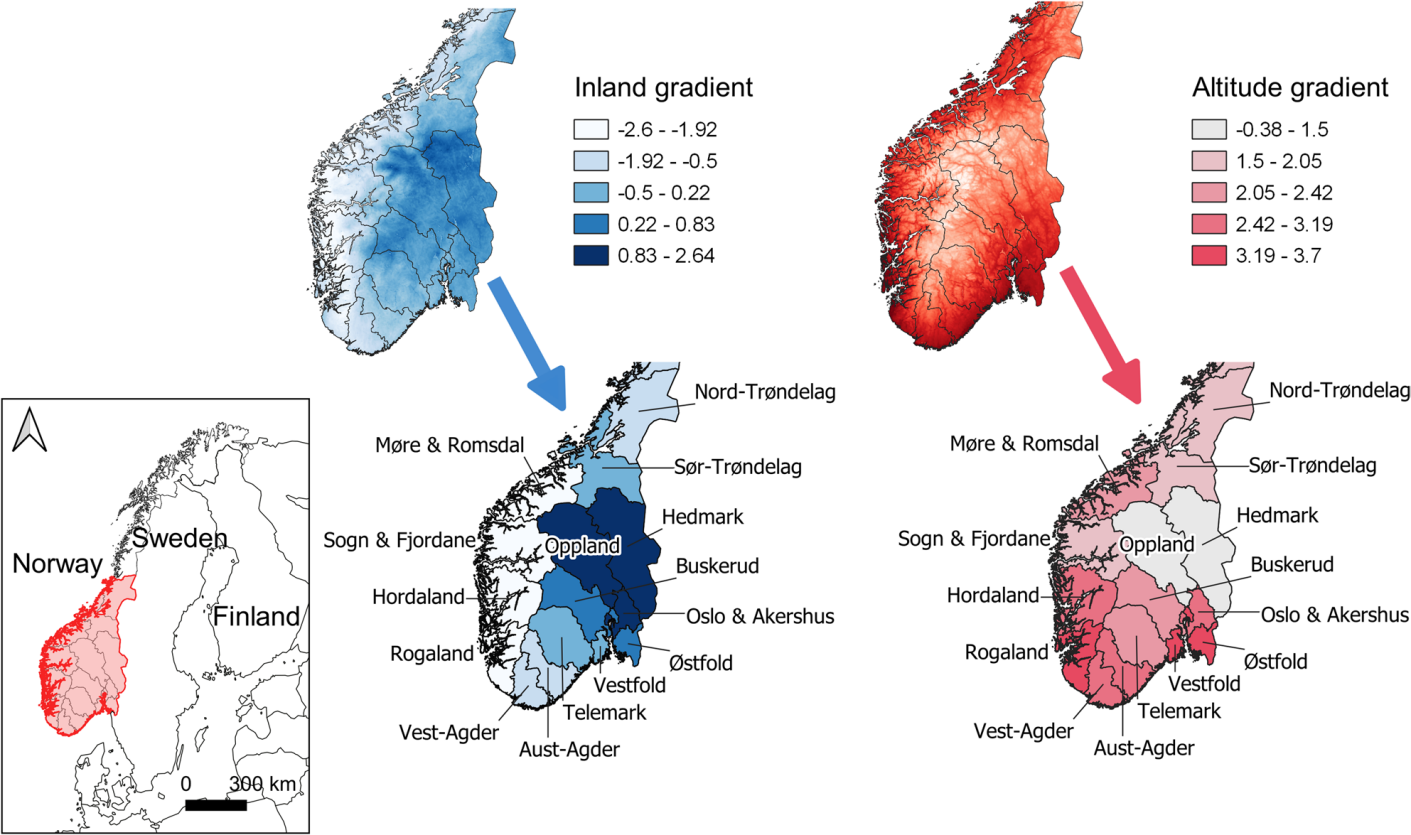
The classification of counties in South Norway by *Inland* and *Altitude* gradients. The upper maps depict the gradients as continuous data, highlighting the within county variation. The lower maps show the means for each county based on values from arable land parcels. Increasing values of the *Inland* gradient are associated with distance from coast and cooler winter temperatures. Increasing values of the *Altitude* gradient are linked with lower latitudes, altitudes and warmer summer temperatures. Inset map shows the location of South Norway (red outline) in relation to Scandinavia. Maps created with QGIS v3.18.1 (www.qgis.org) and gradient data from^20^.

The counties in Western Norway, i.e. Møre and Romsdal, Sogn and Fjordane, Hordaland and Rogaland, exhibited the lowest scores on the *Inland* gradient (close to the coast with warmer winter temperatures) and those in the south east, such as Oppland, Hedmark and Oslo and Akershus, had the highest scores (continental “inland” climate with cooler winter temperatures; Fig. 2). Oppland and Hedmark in the east scored lowest on the *Altitude* gradient indicating tendencies for relatively high altitudes and cooler summer temperatures. The southernmost counties tended towards higher levels of this gradient, with generally warmer summer temperatures and terrain more suited to arable farming. However, it should be noted that some counties had a wide range of scores for each gradient (Supplementary Table S4). This is because counties such as Oppland and Sogn and Fjordane are topographically diverse and arable land is patchily distributed along fjords and throughout glacial valleys, where they experience a range of temperatures and precipitation levels over small spatial scales.

### Yield, weather and bioclimate

**Figure 3:**
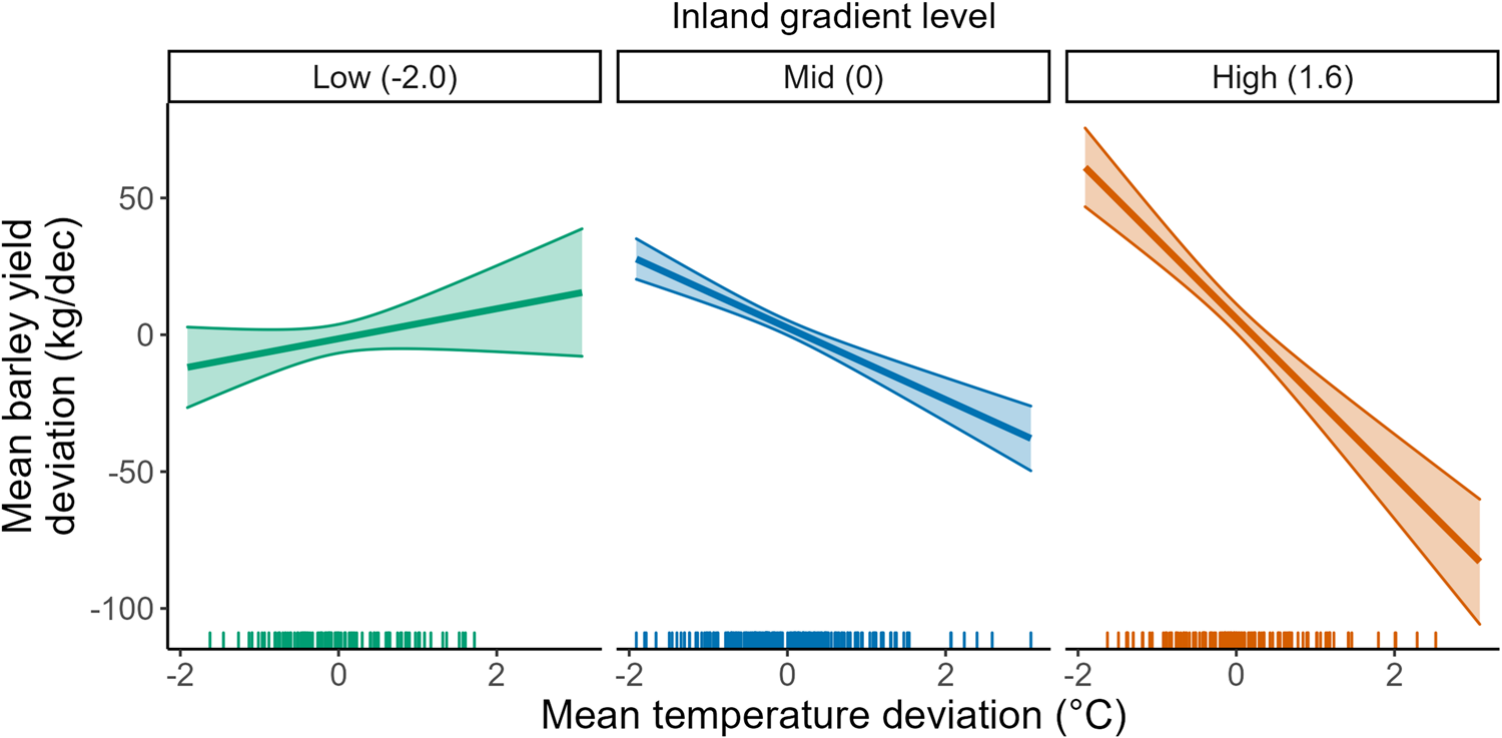
Predicted relationship between mean temperature deviation and mean barley yield deviation for varying levels of the *Inland* gradient. The three graphs are drawn at the 10th, 50th and 90th percentile for the *Inland* gradient. Higher values for this gradient are associated with distance from the coast and cooler winter temperatures. Regression lines show the predicted yield deviation from the LMM when all other predictors are held constant at mean values. Shaded areas are ± 1 SE.

The mixed-effects models performed poorly in general, with r-squared values below 0.1 (Table 1). There was no association between weather variables or bioclimate gradients and wheat yield, both for the linear mixed effects model with all data included (Table 1), and the model with the outlying year 2018 removed (Supplementary Table S5). However, there was a significant interaction between the *Inland* gradient and mean temperature in the barley yield model (Table 1, Fig. 3), which was consistent with the outlying year removed. This interaction suggests that the association between barley yield and mean temperatures is strongly negative in counties such as Hedmark and Oppland, with inland climates and cooler winter temperatures. The negative association is also likely to occur in counties with moderate *Inland* gradient scores, such as Buskerud, Nord-Trøndelag, Sør-Trøndelag, Oslo & Akershus, Østfold, Telemark and Vestfold, but to a lesser extent. Conversely, this relationship was not apparent for counties with low scores on this gradient, such as those in the west of the country. The influence of temperature may even be positive in these counties, although the large standard error around the predicted relationship suggest high uncertainty. The linear mixed effects model for barley yield also suggests a negative relationship between yield and precipitation, but only when 2018 was excluded (Supplementary Table S5). For potato yield, there was only a consistent negative relationship between precipitation and yield both with (Table 1) and without the outlying year (Supplementary Table S5).

**Table 1:** Coefficients and 95% confidence intervals for linear mixed models with detrended yield of each crop as response.

	Wheat		Barley		Potato	
	Estimate	95% CI	Estimate	95% CI	Estimate	95% CI
Intercept	2.96	[− 36.46, 42.37]	2.11	[− 14.58, 18.80]	12.36	[− 78.22, 102.95]
Average temperature (detrended)	− 10.29	[− 60.35, 39.77]	− 5.34	[− 27.67, 17.00]	27.96	[− 91.59, 147.50]
Precipitation (detrended)	− 0.12	[− 0.73, 0.49]	− 0.25	[− 0.52, 0.02]	**− 1.98**	**[− 3.38, − 0.58]**
Inland	2.49	[− 15.98, 20.95]	2.06	[− 2.88, 6.99]	1.64	[− 20.40, 23.68]
Altitude	− 0.42	[− 12.84, 12.00]	0.22	[− 6.33, 6.77]	− 3.82	[− 40.08, 32.44]
Average temperature x Inland	− 11.08	[− 34.42, 12.27]	**− 9.53**	**[− 16.30, − 2.77]**	− 22.77	[− 53.13, 7.58]
Precipitation x Inland	− 0.10	[− 0.39, 0.19]	− 0.01	[− 0.08, 0.06]	− 0.12	[− 0.41, 0.16]
Average temperature x Altitude	− 4.00	[− 20.19, 12.20]	− 3.25	[− 12.06, 5.56]	− 30.77	[− 79.35, 17.81]
Precipitation x Altitude	0.03	[− 0.16, 0.23]	0.03	[− 0.08, 0.13]	0.34	[− 0.19, 0.88]
Random slopes	**0.19**	**[0.06, 0.56]**	**0.22**	**[0.13, 0.38]**	0.00	[0.00, Inf]
R-squared	0.06		0.07		0.05	
RMSE	84.2		56.7		360.5	
LOOCV RMSE	95.3		65.2		372.3	

Values in bold indicate those estimates with confidence intervals that do not encompass zero.

*RMSE* root mean squared error, *LOOCV* leave-one-out cross validation.

### Exploring the effects of monthly aggregated climate data

**Figure 4:**
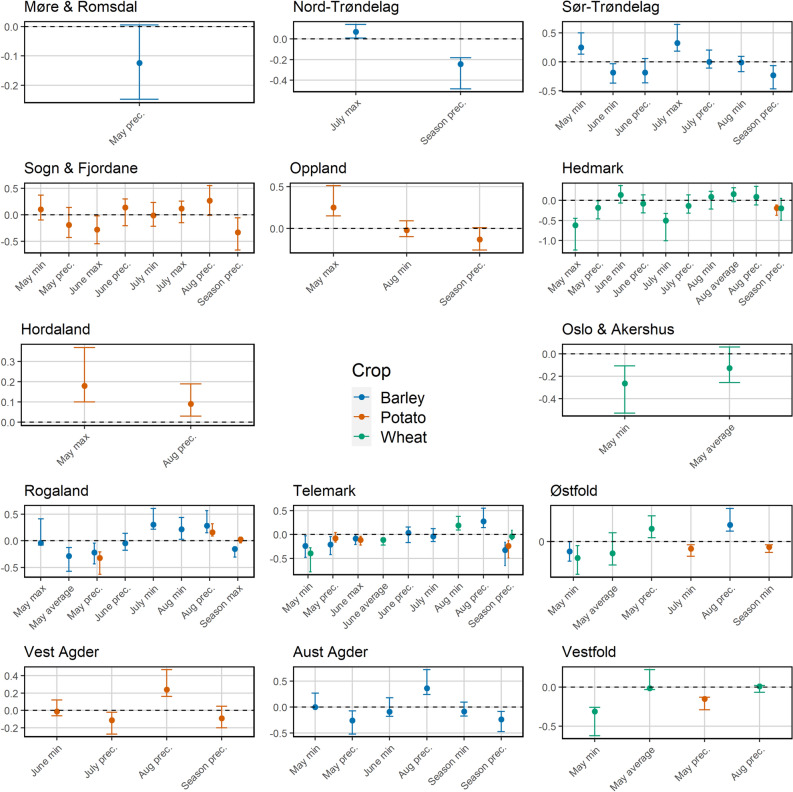
Standardised regression coefficients (all y-axes) from county-level crop models using LASSO, with bootstrapped 95% confidence intervals. The coefficients shown are those that were not reduced to zero and are therefore considered to be the most important predictors of detrended yield. There is no graph for Buskerud County because all predictors were reduced to 0 for all crops. (Abbreviations: *min* minimum temperature, *max* maximum temperature, *prec* precipitation). Coefficients and confidence intervals for these models can be found in Table S7.

The LASSO models generally performed better than the linear mixed effects models for most counties, with deviance ratio values (the fraction of deviance explained) ranging from 0.07 to 0.60 (Supplementary Table S6). The procedure also identified a range of predictors associated with yield of the three crops, and in many cases the growing season variables were not selected at all (Fig. 4) indicating that for many counties these averaged or pooled variables are inadequate predictors of yield. For barley and the association with temperature, the LASSO results were not consistent with the mixed effects model. The only counties with strong links (Confidence Intervals not overlapping 0) between temperature and barley yield were Sør-Trøndelag (May minimum and July maximum) and Rogaland (July minimum, May average and growing season maximum), which both showed relationships in the opposite direction to those estimated by the mixed effects model (Fig. 4). Conversely, the results do support the mixed model (with 2018 removed) findings in relation to precipitation in some counties: there were strong negative links reported for Nord- and Sør-Trøndelag (growing season precipitation), Aust-Agder, Rogaland and Telemark (May and growing season precipitation), but positive for links between yield and August precipitation for Aust-Agder, Rogaland, Telemark and Østfold.

For wheat, there was a strong negative link between May temperatures and yield in the southern and eastern counties Hedmark (May maximum, also July minimum), Oslo and Akershus, Telemark, Østfold and Vestfold (minimum). In addition, wheat yield in Telemark was also negatively associated with June average temperatures, and positively linked to August minimum temperatures. By contrast, potato was positively associated with May temperatures in Oppland and Hordaland (maximum), and negatively linked to temperatures later in the growing season in some other counties, such as Telemark (June maximum) and Østfold (July and growing season minimum). This crop also appeared to be influenced by precipitation differentially throughout the country, as there were positive links for the south western counties Hordaland, Rogaland and Vest-Agder (August), and negative links in Rogaland and Vestfold (May), Vest-Agder (July) and Telemark (growing season). It is also noteworthy that the omission of the extreme year 2018 had varying effects on the results. In some counties the difference in deviance ratio was small, but in others (Hedmark and barley, Buskerud and potato, Sogn and Fjordane and potato; Supplementary Tables S6 and S7) the year appeared influential to the result, indicating sensitivity of this approach to extreme weather.

## Discussion

This study analysed 40 years of wheat, barley, and potato yield against climate data for the first time in Norway to assess the likely effects of recent climate change (as measured by the change in temperature and precipitation), given regional differences in bioclimate. While temperature and precipitation at our selected weather stations have increased in Norway since 1980 in line with the evaluation of the Norwegian Climate Service Centre^21^, all three crop yields exhibited a range of trends at the county level. We demonstrated that although it was possible to fit a valid country-wide model to yield data, the poor fit of the models, and the varying LASSO model results demonstrate that the impacts of local growing season weather are unlikely to be consistent throughout the country. The wide variety of influential monthly variables to county-level yield changes make generalisation for this diverse country difficult and unsuitable, but provide important and novel insights into the importance of local topography to yield-weather relations.

### Wheat

Wheat yield in Norway has remained relatively constant over the previous 40 years, despite improvements in crop varieties, agricultural inputs (fertilizers, insecticides, herbicides, and fungicides), and mechanization that should, in theory, result in a significant increase^48^. Technology based improvements may have been offset by diminishing wheat production profitability since the 1980s due to lower wheat grain prices and higher input and machinery prices^49^. Further, due to environmental concerns, the use of chemical fertilizers and plant protection have been reduced by government restrictions in Norway over the previous two decades^15^. The introduction of new varieties likely had a relatively small impact as the highest yielding Zebra variety was only launched in 2002, while other varieties have shown negligible yield improvements^15^.

The mixed effects model for wheat performed poorly and did not identify any significant growing season climate trends. This is likely to be because the use of growing season weather variables is inappropriate for this crop, and that the model could not account for county-level differences adequately. It is also possible that our growing season variables were too crude, not adequately matching the varying crop calendars among years and counties. The influence of local factors was supported by the LASSO results, where increasing early season temperatures were negatively related to wheat yield in several eastern and southern counties. According to our bioclimatic summary values, these counties typically have a slightly continental climate with warm summer temperatures and are among the warmest areas in Norway where agricultural production is focussed. In other European countries, increasing temperatures since 1980 have also been linked to considerable yield decreases. In Austria, for example, process-based crop simulation models found that wheat yield was reduced by 6% for each degree that temperature increased above optimal levels^50^, and in Denmark, an increase of 1 °C reduced the wheat dry matter yield by 3.5%^25,51^. While peak growing season temperatures in Norway were rarely higher than the optimal 16–22 °C during the study period^52^, rising temperatures in May are important to the shoot emergence and stem elongation stage of wheat growth. As May temperatures often had significant negative effects on yield in our LASSO models, we hypothesise that warmer temperatures in this month may impact the early growth stages in some years. At this time, high temperatures can cause membrane thermo-instability, lower leaf chlorophyll concentration, and reduced photosynthesis, resulting in wheat growth reduction and crop dieback^14,53^. Temperatures in other months and growth stages are also important to wheat yield^54,55^, but the lack of significant effects for these months in this study suggests that temperature changes in Norway during these periods have not yet reached threshold levels.

The lack of growing season and monthly precipitation effects on wheat yields (apart from the positive effect of May rainfall in Østfold) also indicates that increasing precipitation has not yet reached problematic levels in Norway. Further, this is supported by findings in other studies about the low sensitivity of wheat to excessive precipitation and high sensitivity to drought conditions^56,57^. In Norway, wheat is a rainfed crop, but farmers may irrigate during particularly dry summers, so that water is not a limiting factor as in southern European countries^28^. Excessive rainfall was apparently not problematic to wheat yield in our dataset, but we have not been able to assess effects across finer temporal- or spatial-scales, or to assess the efficacy of local adaptation measures to changing weather variables.

### Barley

Barley yield trends were diverse among the counties over the study period, but at the country scale yield has been relatively consistent^58^, in line with other Nordic countries^15^. As with wheat, farmers faced restrictions imposed by fertilizer and chemical plant protection regulations during the 1980s, and tended to respond with less intensive (decreased) production^59^. Introduced varieties have had a favourable impact on barley yield in Norway^15^, with 25% yield increases attributed to the use of varieties resistant to diseases such as scald, net blotch, and ramularia leaf spot^60^.

In relation to the effects of recent climate change, we expected that rising temperatures in Norway would positively impact yields in our temperature limited region. The linear mixed effects model suggested that this may be the case with a tendency towards increased yields in the western counties, but a more dramatic decline in regions with a continental climate. Our LASSO models did not entirely support this, probably because weather variables at finer temporal scales are more important to county-level yield trends. However, previous studies do suggest that this finding is plausible. For example, in southern Finland, increasing temperature reduced barley yield considerably even with adjusted earlier sowing^24^, and temperature rises in European countries reduced average barley yields by 3.8%^61^. Conversely, in cooler, oceanic climates, recent warming may improve the possibility of producing barley^59,62^, as rising temperatures increase growing degree days, and therefore, barley yield^63^. In similar climates in Finland, a 1 °C increase resulted in a 10-day increase in the growing season, lower probability of frost, and increases in the rates of barley growth and development^17^.

The LASSO models also suggested that precipitation effects were important for barley production, and previous work in Norway has suggested a negative impact on the quantity and quality of harvested barley^15^. Barley lacks a physiological mechanism to deal with excess moisture^64^, and excessive rainfall and anaerobiotic soil conditions can reduce yield by 12–20%^65^. Humidity can also increase the incidence of disease infestations, reducing grain size and yield in the process^15^. Nevertheless, the impact of precipitation was not universal in this study. The negative seasonal link was strong for the northernmost counties, but in some southern counties the link was positive in August. This is surprising as late season excessive precipitation is often detrimental to barley^65,66^. This may suggest an interaction with temperature, if warm temperatures in the south combine with increased late season rain synergistically to improve growth. However, to confirm this we would need to study the patterns at finer spatial and temporal scales. We may also have expected that higher temperatures at the beginning and later part of the growing season (May and July) in western regions would help extend the growing season^67^, but this pattern was only found for two counties. Similarly, a detrimental effect of warm May (germination to double ridge stage) temperatures has been found in numerous studies^68–70^, but not in our study.

### Potato

As with barley, there was a wide range of potato yield trends, but with most being positive probably in part due to new varieties, which expanded from 20 to 45 varieties in the study period^71^. This period has also seen an increase in the mechanisation of potato harvest, and other trends such as an increase in farm size and concentration of production around factories and in regions where harvest with machines is most efficient^72^. The spread of pests and diseases also affected potato yields, with potato leaf hopper and aphids prevalent in southern counties and the spread of late blight in mid-Norway^73^.

The large number of varieties of potato available probably makes the crop type tolerant to a wide range of temperatures, and this may explain why increasing temperature had no general association with potato yield in our mixed effects model, as shown in previous work^27,72–75^. However, there were some county-level patterns, with negative links in some southern counties where temperatures may have regularly exceeded the optimum (13–24 °C), which can lead to 6–10% potato yield loss^18,27^. Increased minimum temperatures can also slow or inhibit tuberization in June and tuber bulking in July^75,76^, and can promote the spread of pests and diseases^77^. Positive associations between May temperature and yield in Oppland, are exemplified by the data from 2018 when Oppland had the highest potato yield in the hottest and driest year of the 40 year study period. Generally mild early-season temperature increases in otherwise temperature-limited regions can result in extended frost-free growing seasons, and a reduction in late spring frost damage^74,75,78^. However, the limit of these findings to two counties prevents generalisation of these results, again suggesting that finer scale study is required to identify the most important factors and periods in potato yield.

In general, potato was found to be sensitive to precipitation, in line with studies that reported the highest tuber yield loss in years with excess rainfall^79,80^. Potato is more susceptible to water stress than most crops due to a relatively shallow rooting system and inefficient water transport system^81^. This sensitivity also appeared in monthly variables in some counties, particularly in the south west in May during the sprouting stage, and July during tuber bulking. However, wet late season periods when potatoes are reaching maturity tend to be positive for yield in some counties. As with barley, this is perhaps surprising as excessive late season rain is often destructive for potatoes^78,82^. These positive effects may indicate that rain in Norway has not reached “excessive” levels in some counties, but may also suggest that finer scaled temporal patterns are being masked by our arbitrary monthly divisions. For example, if late August precipitation is high after a good, and early harvest, our models are likely to indicate positive effects as artefacts. We therefore suggest that future research aims to conduct finer-scaled studies, including the use of crop- and farm-specific growing season, to provide more accurate predictions and improve the validity of weather-yield relations.

### Implications

Understanding the effect of climate change on crop yields is critical for achieving food security goals, as well as for policymakers developing food production programs, agricultural development initiatives, and climate-related adaptation measures^83^. However, previous studies have shown that attempting to generalise model findings and yield predictions across regions can be problematic^12,18,76^. In this study, we also demonstrate the difficulty in generalising yield and climate patterns across a hugely diverse country at the northern limit of arable farming. Country-level statistical models that account for county-level climatic differences had low explanatory power, and revealed some general results that were not always supported by finer-scale (LASSO) models. Furthermore, even county-level models demonstrate evidence of masking finer grained responses of yield to climate. For example, the LASSO models were sensitive to the removal of the extreme year (2018) and used crude monthly estimates of weather variables from weather stations that varied in distances from actual farms. Furthermore, we used total yield data for each county, but these data are reported from farms with a mix of soil types, management factors and a wide range of climate conditions.

While it appears that each county should use a separate range of weather predictors to make assessments of future adaptation to climate change, we suggest that yield and weather data are required at finer levels of spatial and temporal detail to make meaningful progress in this endeavour. Although this is no guarantee for modelling success, we can tentatively suggest that some counties could design climate adaptation strategies based on climate risks occurring at the most critical crop growth stages, such as using certain crop varieties, adjusting sowing dates and/or adapting irrigation regimes. However, it has not been possible to make recommendations for specific adaptation measures. More detailed studies at county or regional levels may be able to address some of the shortcomings of our study, such as accounting for variable crop calendars (rather than the static crop calendar used here) and local adaptation efforts, or the use of more appropriate local climate indices such as frost/chill days, growing degree days, heat days and the length of the growing season^78,84^. Some of the data we used are also likely to have affected model performance. Arable land parcels taken from the CORINE land cover map were not crop specific, and precipitation sums and average temperatures are rather simple metrics that do not always reflect other important growth conditions such as rainfall intensity, prolonged dry periods and temperature variability.

In general, it seems that the three crops studied here are not under immediate threat from climate change in Norway, and that other factors such as agricultural policy, management practices, and crop variety selection are more significant. Climate change may rather present arable opportunities in some counties to grow crops that require higher temperatures, or to increase barley and potato production areas. At the very least, our results suggest that the diversity of the agricultural settings in Norway may help to reduce the impact of climate change on the agricultural industry, with reductions in some crops potentially compensated by increases in others. However, given the projected increase of temperature and precipitation in Norway until 2100 and the observed crop yield trends in this study, climate change may still be a credible threat to wheat, barley, and potato productivity in some counties of Norway in the future, and close county-level monitoring is required should extreme years such as 2018 occur more frequently in the future.

## Supplementary Information

Supplementary Information.

Supplementary Table S7.

## Data Availability

The data used in this article are publicly available from Statistics Norway: https://www.ssb.no/en/statbank/table/07479 and the Norwegian Meteorological Institute: https://frost.met.no/howto.html.
