# Supplementary material for: Impacts of recent climate change on crop yield can depend on local conditions in climatically diverse regions of Norway
Source: Sci Rep. 2023 Mar 3;13:3633. doi: 10.1038/s41598-023-30813-7 (PMC9984402; doi:10.1038/s41598-023-30813-7)
Supplement: Supplementary file 1 — Supplementary Information. [file 41598_2023_30813_MOESM1_ESM.docx]

**Supplementary information**

**Table S1:** Wheat, barley, and potato area (decare) and percentage in each county in 2018. Note that Trøndelag comprises Sør-Trøndelag and Nord-Trøndelag.

| County | Agricultural area  (decare) | Wheat  (decare) | Wheat  % | Barley  (decare) | Barley  % | Potato  (decare) | Potato  % |
| --- | --- | --- | --- | --- | --- | --- | --- |
| Total | 8977512 | 586496 | 6.5 | 1477618 | 16.45 | 111856 | 1.2 |
| Østfold | 727136 | 190987 | 26 | 190994 | 26 | 4708 | 0.64 |
| Oslo & Akershus | 756284 | 99853 | 13 | 237977 | 31 | 5921 | 0.78 |
| Buskerud | 508924 | 66708 | 13 | 67961 | 13 | 2913 | 0.57 |
| Hedmark | 1055978 | 59519 | 6 | 339313 | 32 | 47656 | 4.51 |
| Oppland | 1002326 | 16838 | 2 | 139431 | 14 | 8777 | 0.87 |
| Vestfold | 403335 | 119915 | 30 | 57988 | 14 | 14351 | 3.55 |
| Telemark | 245677 | 20782 | 8 | 16789 | 7 | 1719 | 0.69 |
| Aust-Agder | 112711 | 241 | 0.01 | 1698 | 2 | 1946 | 1.72 |
| Vest-Agder | 188592 | 0 | 0 | 1957 | 1 | 912 | 0.48 |
| Rogaland | 996774 | 1118 | 0.01 | 18411 | 2 | 5526 | 0.55 |
| Hordaland | 408335 | 0 | 0 | 174 | 0.01 | 64 | 0.01 |
| Sogn & Fjordane | 425845 | 0 | 0 | 0 | 0 | 922 | 0.21 |
| Møre & Romsdal | 539979 | 0 | 0 | 9764 | 2 | 1856 | 0.34 |
| Trøndelag | 1605616 | 10535 | 1 | 395161 | 25 | 14585 | 0.90 |

**Table S2**: Number of years of available crop yield data for each county in southern Norway 1980–2019 (full time series = 40 years).

| County | Wheat | Barley | Potato |
| --- | --- | --- | --- |
| Aust-Agder | 14 | 32 | 40 |
| Buskerud | 39 | 39 | 39 |
| Hedmark | 40 | 40 | 40 |
| Hordaland | 0 | 6 | 40 |
| Møre og Romsdal | 4 | 30 | 40 |
| Nord-Trøndelag | 26 | 38 | 38 |
| Oppland | 40 | 40 | 40 |
| Oslo & Akershus | 40 | 40 | 40 |
| Østfold | 40 | 40 | 40 |
| Rogaland | 11 | 37 | 40 |
| Sogn og Fjordane | 0 | 3 | 40 |
| Sør-Trøndelag | 14 | 38 | 38 |
| Telemark | 36 | 40 | 40 |
| Vest-Agder | 5 | 33 | 40 |
| Vestfold | 40 | 40 | 40 |


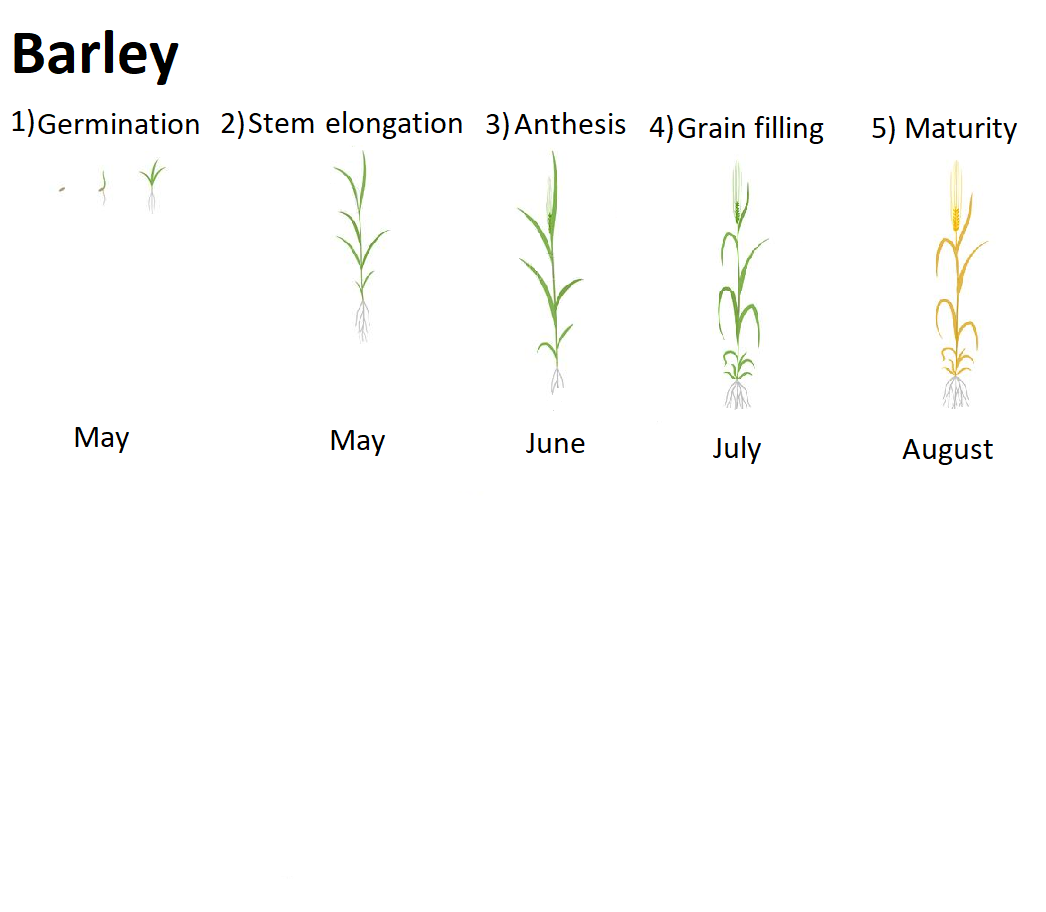

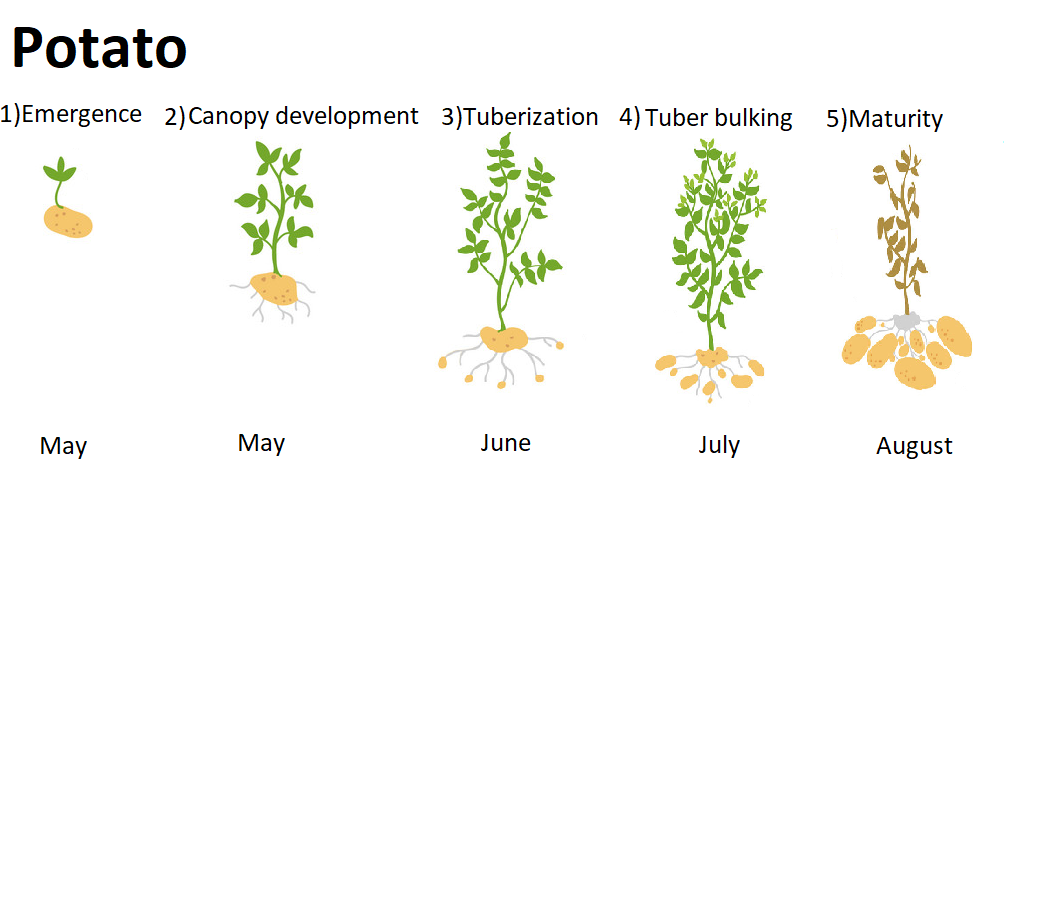

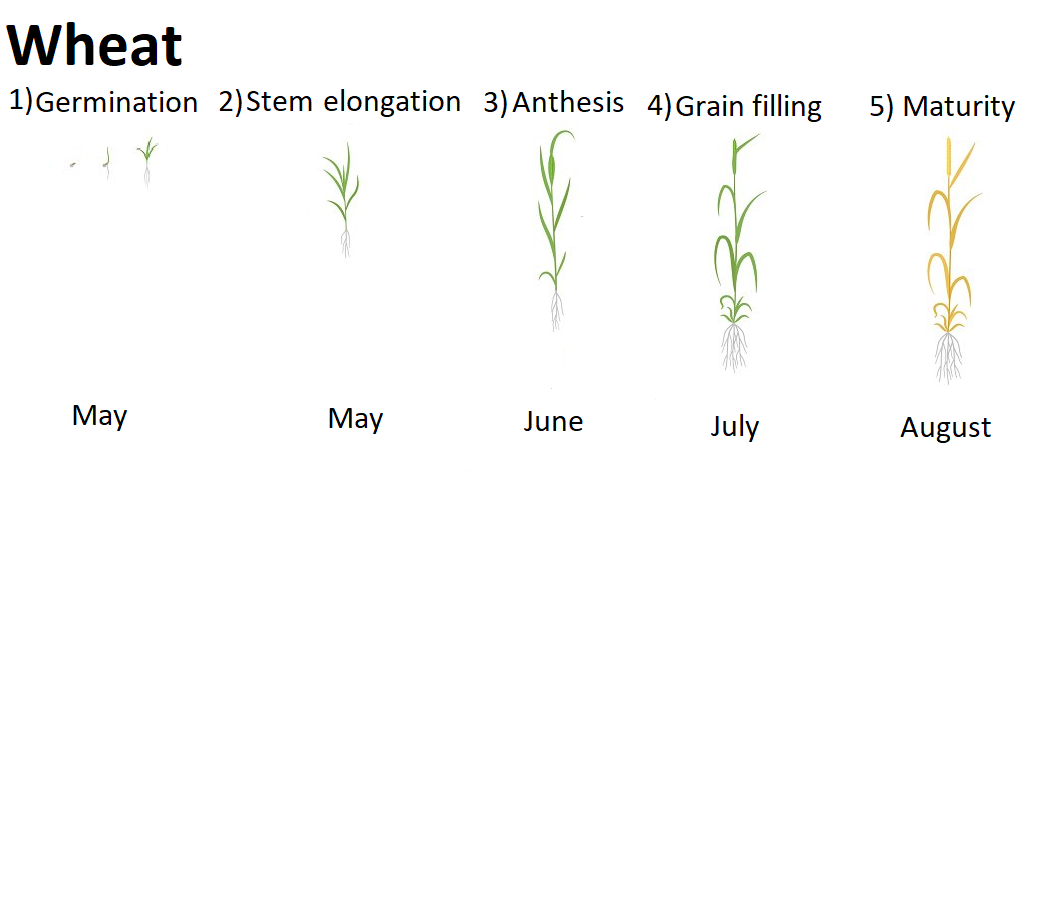


**Figure S1:** Phenological stages of the three focal crops and the months of the growing season that they generally occur in Norway

**Table S3.** Information of the selected meteorological stations for climate data in each county.

| County | Station with temperature and precipitation data | | Station with precipitation data | | Station with temperature data | |
| --- | --- | --- | --- | --- | --- | --- |
|  | **Name** | **Number** | **Name** | **Number** | **Name** | **Number** |
| Østfold | Rygge | SN17150 | Strømsfoss | SN1650 |  |  |
|  |  |  | Fløter | SN17500 |  |  |
| Oslo & Akershus | Blindern | SN18700 | Skedsmo | SN4260 |  |  |
|  | Ås | SN17850 | Eidsvoll Verk | SN11120 |  |  |
|  | Asker | SN19710 |  |  |  |  |
| Buskerud | Kongsberg | SN28380 | Hole | SN20250 |  |  |
|  | Blindern | SN18700 |  |  |  |  |
| Hedmark | Kise | SN12550 | Nord-odal | SN5350 |  |  |
| Oppland | Kise | SN12550 | Biri | SN11900 |  |  |
| Vestfold | Melsom | SN27450 | Sandefjord | SN27600 |  |  |
|  | Blindern | SN18700 | Hedrum | SN27800 |  |  |
|  |  |  | Notodden | SN30530 |  |  |
|  |  |  | Høidalen I | SN32780 |  |  |
|  |  |  | Kviteseid | SN32850 |  |  |
| Aust-Agder | Nelaug | SN36560 | Eikeland | SN35090 |  |  |
|  | Landvik | SN38140 |  |  |  |  |
| Vest-Agder | Kjevik | SN39040 | Tonstad | SN42810 |  |  |
|  | Lista Fyr | SN42160 |  |  |  |  |
| Telemark | Nelaug | SN36560 | Eikeland | SN35090 |  |  |
|  | Landvik | SN38140 |  |  |  |  |
| Rogaland | Sola | SN44560 | Egersund | SN43360 |  |  |
|  | Sauda | SN46610 | Karmøy | SN47240 |  |  |
| Hordaland | Sauda | SN46610 | Hatlestrand | SN50150 | Flesland | SN50500 |
|  | Takle | SN52860 | Eikemo | SN47820 |  |  |
| Sogn og Fjordane | Takle | SN52860 | Aurland | SN53700 |  |  |
|  | Sandane | SN58070 | Vik I Sogn Iii | SN53070 |  |  |
| Møre og Romsdal | Fiskåbygd | SN59610 | Sunndalsøra Iii | SN63420 |  |  |
|  | Tafjord | SN60500 | Sæbø | SN59900 |  |  |
|  | Vigra | SN60990 |  |  |  |  |
| Sør-Trøndelag | Værnes | SN69100 | Løksmyr | SN68270 | Sula | SN65940 |
|  | Ørland Iii | SN71550 |  |  |  |  |
| Nord-Trøndelag | Værnes | SN69100 | Otterøy | SN75020 |  |  |
|  | Snåsa - Ki | SN70850 | Buran | SN69960 |  |  |

**Table S4:** Mean values and measures of spread of the two climate gradients for each county.

|  | Inland | | | |  | Altitude | | | |
| --- | --- | --- | --- | --- | --- | --- | --- | --- | --- |
| County | **Mean** | **sd** | **Minimum** | **Maximum** |  | **Mean** | **sd** | **Minimum** | **Maximum** |
| Aust-Agder | -0.87 | 0.25 | -1.36 | -0.27 |  | 2.71 | 1.01 | 0.64 | 4.27 |
| Buskerud | 0.83 | 0.64 | -0.29 | 2.31 |  | 2.19 | 1.10 | -1.29 | 3.36 |
| Hedmark | 1.68 | 0.53 | -1.14 | 2.96 |  | 1.43 | 1.19 | -1.49 | 3.21 |
| Hordaland | -2.60 | 0.92 | -3.86 | -1.07 |  | 2.44 | 0.99 | 0.38 | 4.13 |
| More & Romsdal | -1.98 | 0.80 | -3.60 | 0.02 |  | 2.32 | 0.76 | 0.00 | 3.51 |
| Nord-Trøndelag | -0.51 | 0.68 | -2.39 | 1.46 |  | 2.02 | 0.72 | 0.00 | 3.91 |
| Oppland | 1.63 | 0.50 | 0.35 | 3.10 |  | 0.37 | 1.00 | -1.77 | 2.39 |
| Oslo & Akershus | 0.84 | 0.30 | 0.12 | 1.49 |  | 2.94 | 0.38 | 1.86 | 3.99 |
| Østfold | 0.51 | 0.19 | -2.56 | 0.83 |  | 3.49 | 0.36 | 0.00 | 4.11 |
| Rogaland | -2.28 | 0.63 | -3.64 | -0.85 |  | 3.66 | 0.53 | 2.07 | 4.49 |
| Sogn & Fjordane | -2.56 | 0.91 | -4.23 | 0.22 |  | 1.90 | 0.91 | 0.11 | 4.05 |
| Sør-Trøndelag | -0.45 | 1.08 | -2.33 | 2.23 |  | 1.55 | 1.09 | -0.84 | 3.39 |
| Telemark | 0.03 | 0.35 | -0.73 | 0.67 |  | 2.31 | 1.01 | -0.39 | 3.71 |
| Vest-Agder | -1.88 | 0.48 | -2.42 | -0.64 |  | 3.03 | 1.09 | 0.81 | 4.44 |
| Vestfold | -0.02 | 0.25 | -0.46 | 0.49 |  | 3.70 | 0.29 | 3.11 | 4.08 |

**Table S5**: Coefficients and 95% confidence intervals for linear mixed models with detrended yield of each crop as response, with the extreme year of 2018 removed. Values in bold indicate those estimates with confidence intervals that do not encompass zero. RMSE = Root mean squared error. LOOCV = leave-one-out cross validation

|  | Wheat | | Barley | | Potato | |
| --- | --- | --- | --- | --- | --- | --- |
|  | **Estimate** | **95% CI** | **Estimate** | **95% CI** | **Estimate** | **95% CI** |
| Intercept | 6.51 | [-32.93, 45.94] | 2.92 | [-12.66, 18.50] | 15.02 | [-76.71, 106.75] |
| Average temperature (detrended) | -9.01 | [-58.87, 40.84] | -2.97 | [-24.03, 18.10] | 35.13 | [-89.34, 159.60] |
| Precipitation (detrended) | -0.15 | [-0.74, 0.45] | **-0.30** | **[-0.56, -0.04]** | **-2.19** | **[-3.66, -0.72]** |
| Oceanity | 3.46 | [-14.93, 21.85] | 1.84 | [-2.77, 6.45] | 6.08 | [-16.26, 28.42] |
| Climate | -0.87 | [-13.35, 11.60] | 0.52 | [-5.61, 6.64] | -4.53 | [-41.28, 32.21] |
| Average temperature x Inland | -4.27 | [-27.76, 19.23] | **-6.98** | **[-13.47, -0.49]** | -12.62 | [-44.20, 18.96] |
| Precipitation x Inland | -0.19 | [-0.47, 0.08] | -0.04 | [-0.11, 0.02] | -0.19 | [-0.49, 0.11] |
| Average temperature x Altitude | -1.03 | [-17.19, 15.13] | -1.35 | [-9.80, 7.10] | -32.29 | [-83.26, 18.69] |
| Precipitation x Altitude | 0.02 | [-0.18, 0.21] | 0.02 | [-0.08, 0.12] | 0.39 | [-0.17, 0.95] |
| Random slopes | **0.19** | **[0.03, 1.36]** | 0.00 | [0.00, Inf] | 0.00 | [0.00, Inf] |
| R-squared | 0.05 |  | 0.08 |  | 0.05 |  |
| RMSE | 80.9 |  | 54.2 |  | 359.5 |  |
| LOOCV RMSE | 98.5 |  | 65.2 |  | 372.3 |  |

**Table S6:** The deviance ratio for each of the LASSO models, with corresponding values for models without 2018 in brackets. Deviance ratios indicate the fraction of deviance explained by the models.

| **County** | **Wheat** | **Barley** | **Potato** |
| --- | --- | --- | --- |
| Aust-Agder | - | 0.43 (0.43) | 0.00 (0.00) |
| Buskerud | 0.00 (0.00) | 0.00 (0.00) | 0.00 (0.13) |
| Hedmark | 0.60 (0.55) | 0.00 (0.32) | 0.11 (0.25) |
| Hordaland | - | - | 0.13 (0.13) |
| Møre og Romsdal | - | 0.07 (0.07) | 0.00 (0.00) |
| Nord-Trøndelag | 0.00 (0.00) | 0.21 (0.21) | 0.00 (0.00) |
| Oppland | 0.00 (0.00) | 0.00 (0.00) | 0.23 (0.00) |
| Oslo & Akershus | 0.29 (0.21) | 0.00 (0.00) | 0.00 (0.00) |
| Østfold | 0.32 (0.27) | 0.23 (0.00) | 0.10 (0.09) |
| Rogaland | - | 0.50 (0.61) | 0.26 (0.23) |
| Sogn og Fjordane | - | - | 0.40 (0.00) |
| Sør-Trøndelag | - | 0.47 (0.47) | 0.00 (0.00) |
| Telemark | 0.51 (0.47) | 0.44 (0.45) | 0.23 (0.34) |
| Vest-Agder | - | 0.00 (0.00) | 0.00 (0.09) |
| Vestfold | 0.22 (0.15) | 0.00 (0.00) | 0.10 (0.08) |
